# Rapid determination of the total content of oleanolic acid and ursolic acid in Chaenomelis Fructus using near-infrared spectroscopy

**DOI:** 10.3389/fpls.2022.978937

**Published:** 2022-09-02

**Authors:** Jing Ming, Mingjia Liu, Mi Lei, Bisheng Huang, Long Chen

**Affiliations:** ^1^Key Laboratory of Traditional Chinese Medicine Resources and Chemistry of Hubei Province, Hubei University of Chinese Medicine, Wuhan, China; ^2^Xiangyang Central Hospital, Affiliated Hospital of Hubei University of Arts and Sciences, Xiangyang, China

**Keywords:** near-infrared spectroscopy, Chaenomelis Fructus, oleanolic acid, ursolic acid, quantitative model, partial least squares regression, back propagation artificial neural network

## Abstract

Chaenomelis Fructus is a widely used traditional Chinese medicine with a long history in China. The total content of oleanolic acid (OA) and ursolic acid (UA) is taken as an important quality marker of Chaenomelis Fructus. In this study, quantitative models for the prediction total content of OA and UA in Chaenomelis Fructus were explored based on near-infrared spectroscopy (NIRS). The content of OA and UA in each sample was determined using high-performance liquid chromatography (HPLC), and the data was used as a reference. In the partial least squares (PLS) model, both leave one out cross validation (LOOCV) of the calibration set and external validation of the validation set were used to screen spectrum preprocessing methods, and finally the multiplicative scatter correction (MSC) was chosen as the optimal pretreatment method. The modeling spectrum bands and ranks were optimized using PLS regression, and the characteristic spectrum range was determined as 7,500–4,250 cm^−1^, with 14 optimal ranks. In the back propagation artificial neural network (BP-ANN) model, the scoring data of 14 ranks obtained from PLS regression analysis were taken as input variables, and the total content of OA and UA reference values were taken as output values. The number of hidden layer nodes of BP-ANN was screened by full-cross validation (Full-CV) of the calibration set and external validation of the validation set. The result shows that both PLS model and PLS-BP-ANN model have strong prediction ability. In order to evaluate and compare the performance and prediction ability of models, the total content of OA and UA in each sample of the test set were detected under the same HPLC conditions, the NIRS data of the test set were input, respectively, to the optimized PLS model and PLS-BP-ANN model. By comparing the root-mean-square error (RMSEP) and determination coefficient (*R*^2^) of the test set and ratio of performance to deviation (RPD), the PLS-BP-ANN model was found to have better performance with RMSEP of 0.59 mg·g^−1^, *R*^2^ of 95.10%, RPD of 4.53 and bias of 0.0387 mg·g^−1^. The results indicated that NIRS can be used for the rapid quality control of Chaenomelis Fructus.

## Introduction

Chaenomelis Fructus, the dried fruit of *Chaenomeles speciosa* (Sweet) Nakai (Rosaceae), has high application value of food nutrition and medical health care (Hamauzu et al., 2005). The main chemical compositions of Chaenomelis Fructus include flavonoids, triterpenes, phenylpropanoids, organic acids, and tannins (Du et al., 2013; Lyu et al., 2021). According to Chinese Pharmacopoeia 2020 (ChP 2020), the total content of oleanolic acid (OA, C_30_H_48_O_3_) and ursolic acid (UA, C_30_H_48_O_3_) in Chaenomelis Fructus determined by high-performance liquid chromatography (HPLC) shall be no less than 0.50% (5 mg·g^−1^; Chinese Pharmacopoeia Commission, 2020). HPLC is a mature method widely used in Chinese herb content detection but requires cumbersome pretreatment and large amounts of reagents, it is time-consuming, and produces chemical waste (Lu et al., 2022). Therefore, it is vital to develop a rapid, nondestructive, inexpensive, and effective analytical method for the determination of the total content of OA and UA in Chaenomelis Fructus.

Near-infrared spectroscopy (NIRS) is based on the absorption of organic molecules in the spectral region of 780–2,500 nm. This absorption consists largely of combinations and overtones of C-H, O-H, and N-H fundamental frequencies (Zhang et al., 2017). Thus, NIR spectra can reflect the abundant chemical molecular structure information of Chinese herb medicines. At present, NIRS has attracted much attention because of its advantages of fast detection speed, environmental protection, and nondestructive analysis. It can directly analyze solid or powder samples without extraction or purification (Zhang et al., 2019). With the advent of some portable near-infrared spectrometers, NIRS combined with nondestructive acquisition methods like optic fiber probes can realize on-site, real-time, and online measurements in industrial production (Yang et al., 2022). Based on the above advantages, NIRS has been widely used in agriculture, food, medicine, and other research fields (Xue et al., 2011; Silalahi et al., 2016; Kang et al., 2019).

Due to the nature of NIR (overtones and combination bands of vibrational energy levels), it is difficult to directly obtain relevant and useful information from the raw NIR spectra (Luypaert et al., 2007; Zhang et al., 2022). Thus, chemometrics methods are required to mine the information of NIR spectra to extract relevant information and reduce irrelevant information, and establish correction models for qualitative or quantitative analysis (Chen et al., 2019). Partial least squares (PLS) regression is a commonly used quantitative modeling algorithm, which integrates the advantages of principal component analysis, canonical correlation analysis, and multiple linear regression analysis (Chang et al., 2020). It has the advantages of simple modeling, stable performance, and mature application (Bázár et al., 2016). Artificial neural network (ANN) is a popular intelligent chemometric method. It has many abilities including self-learning, self-organizing, strongly fault-tolerating and adapting high non-linear computing (Wang et al., 2007). Back propagation artificial neural network (BP-ANN), the most widely used neural network, is a type of multilayer feedforward neural network trained according to the error back propagation algorithm. It has been applied in various fields combined with NIRS (Liu et al., 2013; Zhang et al., 2017; Bin et al., 2021).

In this study, a rapid quantitative model of the total content of OA and UA in Chaenomelis Fructus was established using NIRS based on the PLS and BP-ANN algorithms, which was expected to provide a rapid and nondestructive technical reference for quality control and market management of Chaenomelis Fructus.

## Materials and methods

### Instruments and software

The content of OA and UA was determined with the HPLC system DIONEX Ultra 3000 and analytical laboratory DIONEX CHROMELEON (Thermo Fisher, America). A Thermo Hypersil BDS C18 (5 μm, 4.6 mm × 250 mm) column was applied. NIR spectra were collected with an MPA FT-NIR spectrometer (Bruker Optics Co., Ltd., Germany) equipped with an Integrating Sphere Module and InGaAs detector, and a tungsten halogen lamp as the light source. The spectra were analyzed using the OPUS 7.5 spectrum analysis software (Bruker), MATLAB R2014a data analysis software (Math-Works, Inc., United States).

### Samples and reagents

A total of 122 batches of samples were collected from Hubei, Anhui, Chongqing province in China. All samples were identified by Professor Keli Chen (College of pharmacy, Hubei University of Chinese Medicine). Each sample was smashed into powder and passed through a 60-mesh sieve. In order to reduce the effect of water content, all samples were dried at 60°C for 24 h. A total of 90 batches of samples (No. 1~90) were randomly selected for the establishment and validation of quantitative model. The remaining 32 batches of samples were used as test set (No. 90~122) to evaluate the model performance and prediction ability.

Standards of oleanolic acid and ursolic acid (Batch No. 110709-201206 and 110742-201421, respectively; purity > 98% for both) were provided by the National Institutes for Food and Drug Control (Beijing, China). Methanol of HPLC grade and pure water were purchased from TEDIA (United States) and WAHAHA (Hangzhou, China). All other solvents and reagents were of analytical grade unless otherwise noted.

### Spectra acquisition

**Figure 1:**
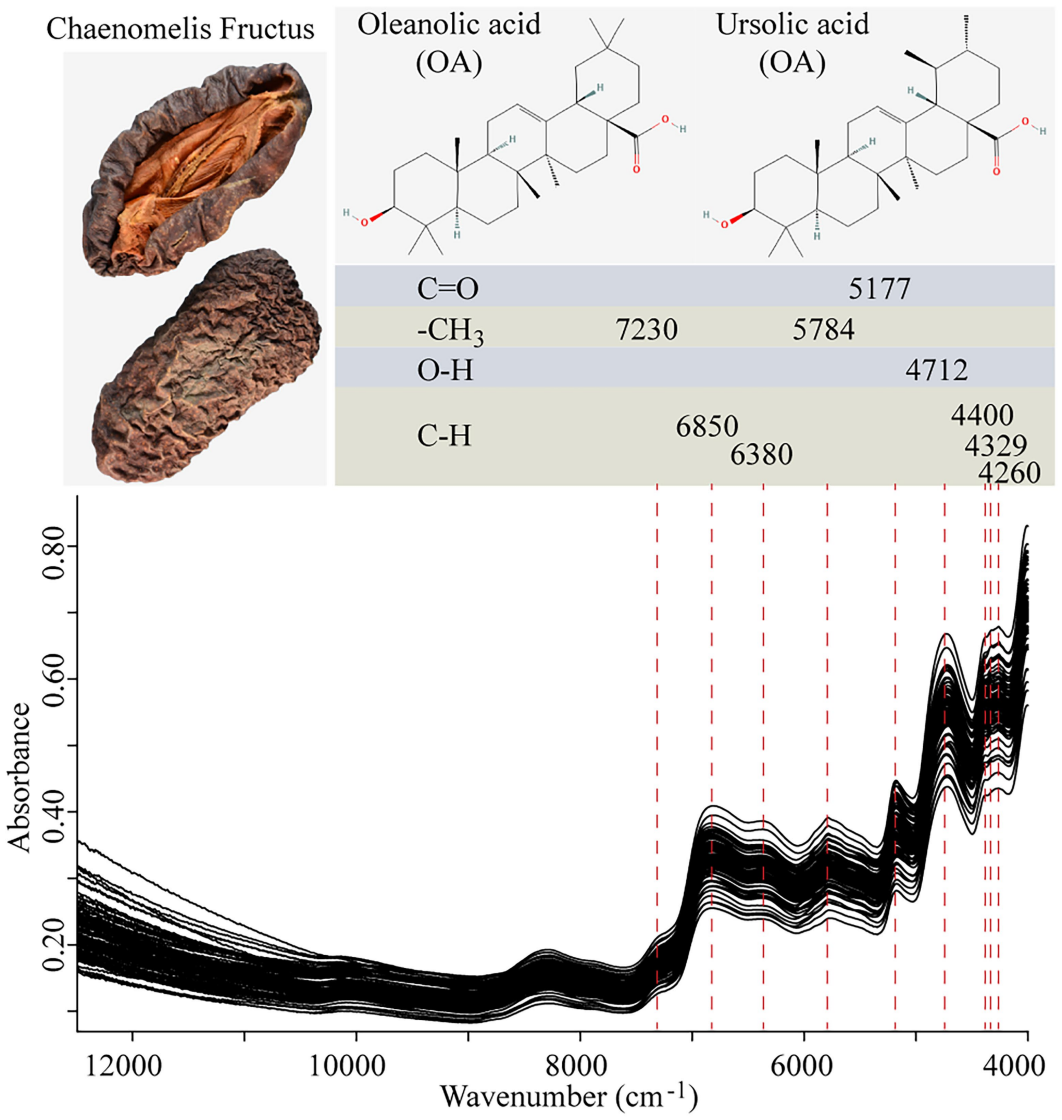
NIR spectra of samples.

NIR spectra were collected with an integrating sphere diffuse reflection module using the MPA FT-NIR spectrometer equipped with an InGaAs detector, and a tungsten halogen lamp as the light source. OPUS 7.5 spectrum analysis software was used in NIRS data acquisition. The spectral data were recorded as the average of 64 scans in the spectral range of 12,500–4,000 cm^−1^ with 0.2 ms per scan, and the spectra were collected at a spectral resolution of 8 cm^−1^, with air absorbance as the reference standard at room temperature (25°C). The spectra of each sample were recorded in triplicate and the average spectrum was used for subsequent analysis. The scanning time of each sample was approximately 38 s. The spectra diagram is shown in Figure 1. It can be seen that the main characteristic peaks are distributed in 9,000–4,000 cm^−1^, so this spectral range is selected as the initial spectral range for modeling.

### Content determination

The content measurement of OA and UA was conducted by HPLC according to ChP 2020 (Chinese Pharmacopoeia Commission, 2020). A quantity of 0.5 g of the sample powder was precisely weighed and extracted in ultrasonic bath with 25 ml of methanol for 20 min. After cooling, the mixture was weighed again, and the lost weight was made up of methanol. The extracted solution was filtered, and the filtrate was used for HPLC analysis. Each sample was repeated twice in parallel according to the above operation, and each extract was injected three times. The average value was taken as the actual content of the samples. Appropriate amounts of oleanolic acid and ursolic acid were precisely weighed, and then methanol was added. The standard solution was prepared to contain 0.1 mg of oleanolic acid and 0.1 mg of ursolic acid in each 1 ml of the solution.

HPLC conditions were as follows: injection volume, 20 μl; column, Thermo Hypersil BDS C18 (250 mm × 4.6 mm, 5 μm); mobile phase, methanol/water/acetic acid/triethy lamine (265:35:0.1:0.05); determination wavelength, 210 nm; flow rate, 1 ml/min; column temperature, 18°C.

### Spectral pretreatment method

Usually, the raw spectrum includes a lot of irrelevant information or noise, which would lead to baseline drift and instability. Therefore, it is very necessary to conduct suitable spectrum pretreatment to eliminate the systematic errors that could be caused by various factors and help increase model performance. In this study, methods such as Savitzky–Golay smoothing (SG), vector normalization (VN), first derivative (FD), second derivative (SD), multiple scattering correction (MSC) and combined pretreatment methods were employed by OPUS to optimize model performance. The Savitzky–Golay smoothing is an averaging algorithm that fits a polynomial equation to the data points. It can improve the smoothness of the spectra and reduce the interference of noise (Chen et al., 2012). The VN is used to normalize a spectrum by an initial calculation of the average intensity value and subsequent subtraction of this value from the spectrum. Then, the addition of the squared intensities is calculated, and the spectrum is divided by the square root of this addition. The MSC performs a linear transformation of each spectrum for it to best match the mean spectrum of the whole set. It mainly eliminates the scattering effect caused by uneven sample size and particle size (Lu et al., 2022). First and second derivatives are used to emphasize pronounced but small features over a broad background. It can effectively eliminate baseline and other background interference (Galvez et al., 2013).

### PLS method

The spectral data processed using different pretreatment methods was associated with the content obtained by HPLC (reference value) and the PLS model was established using the QUANT-2 module of OPUS. In order to improve model prediction capacity and robustness, it is often necessary to screen the NIRS characteristic spectrum and eliminate the interferential variables (Lei et al., 2017). In this study, a common method termed synergy interval partial least squares (SiPLS) is used to screen the characteristic spectral bands. The spectra are split into a variety of intervals, the combinations of which are used to develop PLS models. By comparing the performance of the regression model established by each interval or interval combination, the optimal spectrum range can be screened out (Kang et al., 2015).

The PLS models were validated and evaluated using the internal cross-validation and external validation method. During the process of modeling, the calibration set was used for internal cross-validation to validate model performance. The internal cross-validation adopted leave-one-out cross validation (LOOCV), and the root mean square error of internal cross-validation (RMSECV) and coefficient of determination (*R*^2^) were taken to guide the model optimization process. The validation set was used for external validation to evaluate the model, with the root mean square error of prediction (RMSEP), *R*^2^, and residual predictive deviation (RPD) taken as indexes to evaluate prediction ability. Generally, the smaller the RMSECV and the larger the *R*^2^ are, the better the model performance would be; the smaller the RMSEP and the greater the *R*^2^ and RPD are, the stronger the model prediction ability is (Magalhaes et al., 2016). RPD was the ratio of SD of the calibration set to RMSEP. The higher the RPD value, the better the prediction performance of the model. In addition, the optimal rank is also one of main evaluation indexes for the PLS model. An insufficient lower rank will lead to failure in explaining the change of spectrum component concentration and under-fitting of the model. However, excessive rank will lead to decreased model specificity upon component and over-fitting of the model (Zhou et al., 2014). Therefore, rank was screened in the meantime of spectrum screening. The optimal rank value can be obtained when the values of RMSECV and RMSEP are the lowest.

### BP-ANN method

Artificial neural networks are systems simulating the human brain for processing information like new data and knowledge. A complex network connected by a large number of simple processing units is used to simulate the structure and functions of human brain neural networks to process information. They have been widely used in various fields with self-organization, self-learning, robustness, fault tolerance, and non-linear information processing function (Watanabe et al., 2014). The most commonly used BP-ANN is a multilayer feed-forward neural network containing input, hidden and output layers, featuring the forward propagation of signals and backward propagation of errors with strong non-linear modeling ability, which is suitable for solving complex mapping problems (Khan, 2015).

It is difficult to build ANN models using NIRS data directly due to the large amount of spectra data, high data dimensionality, multicollinearity and noise among the data, all of which lead to poor model stability (Sun et al., 2017). Therefore, dimensionality reduction is needed. The PLS dimensionality reduction method was combined with BP-ANN modeling algorithm to construct a PLS-BP-ANN model by using the principal factor data obtained from optimizing the optimal PLS model as the input variables, and the total content determined by HPLC as the output variables of BP-ANN for better model performance. Through internal cross-validation and external validation, the predictive ability of model is evaluated. In terms of the internal cross-validation, root mean square error of prediction (RMSEP_c_) and coefficient of determination (*R*^2^_c_) of the calibration set are taken as indexes to guide model optimization process. While in external validation, root mean square error of prediction (RMSEP_v_), *R*^2^_v_ and RPD of the validation set are taken as indexes to further evaluate the model prediction ability. In addition, to ensure that the optimized models are reliable, the bias between the predicted and reference data was also considered.

## Results and discussion

### Determination of OA and UA contents

The HPLC results of 90 samples for modeling are shown in Supplementary Table 1. The OA content was between 1.9 and 13.4 mg·g^−1^, the UA content was between 0.2 and 5.0 mg·g^−1^, the total content of OA and UA was between 4.2 and 15.3 mg·g^−1^. The samples are representative with a broad content range. 7 samples with the total content of OA and UA below 5 mg·g^−1^ is unqualified, accounting for 7.8% of all samples. A total of 90 samples were randomly divided into calibration set and prediction set in a proportion of 2:1. The variation range, mean, standard deviation (SD) and coefficient of variation (CV) of measured values are shown in Table 1.

**Table 1:** Statistic results of the calibration set and validation set.

Set	Samples	OA (mg·g^−1^)				UA (mg·g^−1^)				Total content of OA and UA (mg·g^−1^)			
		Range	Mean	SD	CV	Range	Mean	SD	CV	Range	Mean	SD	CV
Calibration set	60	1.9–13.4	7.7	2.6	0.34	0.2–5	1.5	1	0.67	4.2–15.3	9.2	2.6	0.28
Validation set	30	2.1–13.2	7.8	2.6	0.33	0.3–3.2	1.5	0.9	0.60	4.4–14.5	9.3	2.7	0.29

### NIR spectral characteristics

NIR characteristic peaks of samples are shown in Figure 1. In the figure, the chemical bonds or groups of OA and UA are matched with their possible characteristic absorption peaks. The peak 5,177 cm^−1^ is induced by the C=O stretch of carboxylic acid and around 7,230 cm^−1^ and 5,784 cm^−1^ can be attributed to the combination absorbance of C-H antisymmetric stretching and C-H bending in -CH_3_. Among other characteristic absorbance peaks are the combination of O-H (4,712 cm^−1^) and C-H (4,400, 4,329, and 4,260 cm^−1^). These results were consistent with other researches (Ma et al., 2019). However, these signals can be caused by ingredients like flavonoids, organic acids, and some other compounds in Chaenomelis Fructus, too. The absorption bands can be attributed to the contributions of multicomponents and the shifts and distortions that result from their interactions. Therefore, the spectral pretreatment and chemometric methods are required to highlight and extract the useful information that is mainly correlated with OA and UA.

### Development of the PLS model

#### Selection of modeling target

The initial modeling spectra of samples were matched with the OA, UA content and the total of both, respectively, to build PLS models. The model validation results are shown in Table 2. The performance and predictive ability of the NIRS quantitative model of OA (M1) and UA (M2) were poor, while that of the model of the total content (M3) were better. It might be unsuitable to consider OA or UA as one single component in modeling since both are pentacyclic triterpenoids with similar chemical structures, their NIRS characteristics interfering with each other. Moreover, the total content of them have been used as a quality control marker of Chaenomelis Fructus, as documented in ChP 2020. Therefore, the total content of OA and UA was selected as modeling target. Subsequent models and optimizations were developed for the total content of OA and UA.

**Table 2:** Modeling results based on different compound.

Model number	Compound	LOOCV		External validation			Rank
		RMSECV (mg·g^−1^)	*R*^2^ (%)	RMSEP (mg·g^−1^)	*R*^2^ (%)	RPD	
M1	OA	1.76	52.00	1.3	72.89	1.97	20
M2	UA	0.92	6.34	0.81	14.05	1.09	6
M3	OA+UA	1.14	80.50	0.73	92.17	3.58	18

#### Optimization of pretreatment

Different pretreatment methods include SG, MSC, FD, SD and combined pretreatment methods were compared to investigate the influences on the performance of PLS model. The optimization results are shown in Table 3. Model M7 has a good modeling effect and the strongest prediction capacity. Thus, MSC was finally selected as the optimal pretreatment method.

**Table 3:** Optimization results of spectra pretreatment in calibration models.

Model number	Pretreatment	LOOCV		External validation			Rank
		RMSECV (mg·g^−1^)	*R*^2^ (%)	RMSEP (mg·g^−1^)	*R*^2^ (%)	RPD	
M4	VN	1.02	84.46	0.71	92.74	3.72	20
M5	FD+SG (17 points)	1.00	85.13	0.84	89.66	3.11	15
M6	SD+SG (17 points)	1.26	76.23	1.03	84.50	2.54	18
M7	MSC	1.02	84.6	0.66	93.67	3.98	19
M8	FD+VN+SG (17 points)	0.99	85.34	0.76	91.60	3.46	14
M9	FD+MSC+SG (17 points)	1.07	82.85	0.71	92.56	3.67	12

#### Selection of NIR characteristic spectral bands

**Figure 2:**
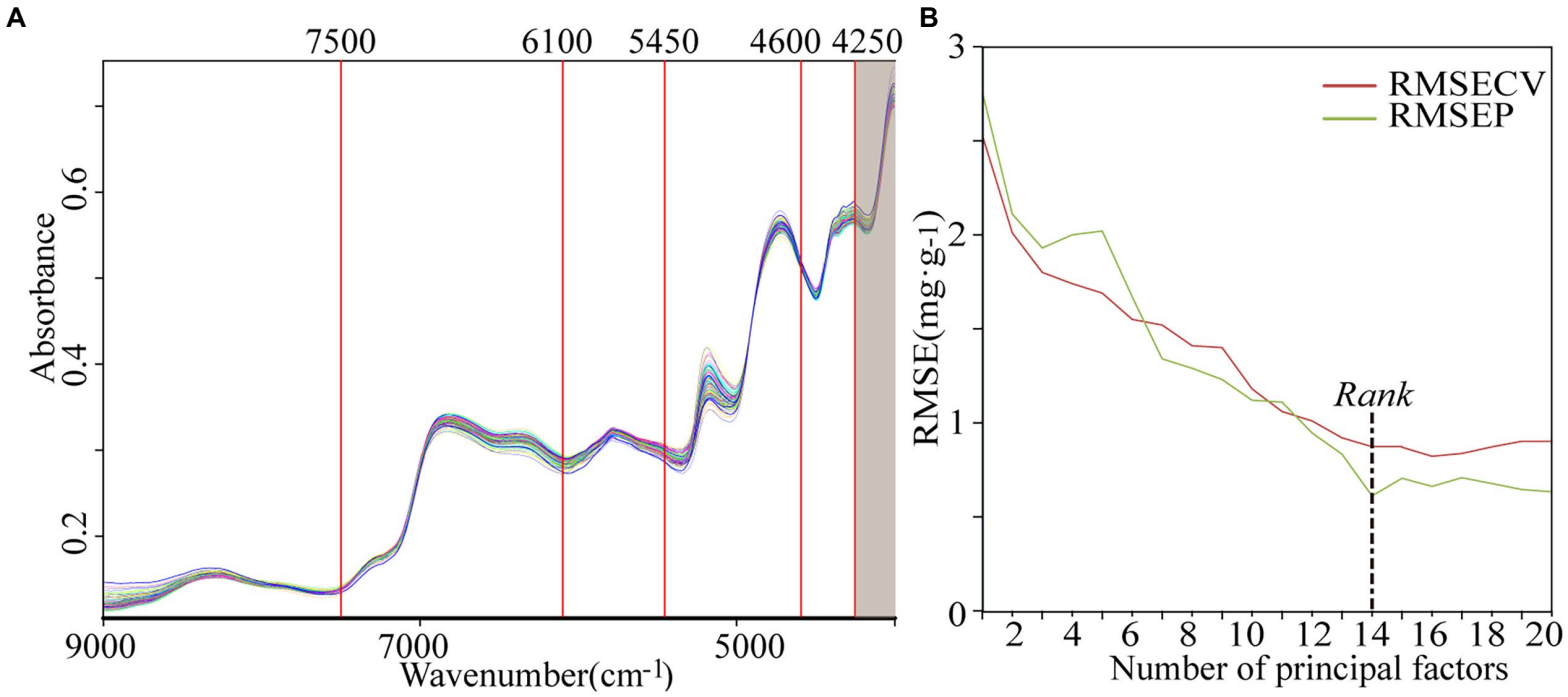
The characteristic interval division **(A)** and correlation curve between number of principal factors and RMSE in the PLS model **(B)**.

The SET function in the QUANT-2 module of OPUS 7.5 software was used for the automatic division of the MSC-treated 9,000–4,000 cm^−1^ spectrum into five characteristic intervals (shown in Figure 2A). The combinatorial screening of the intervals determined the optimal modeling spectrum of 7,500–4,250 cm^−1^. The correlation between the RMSECV, RMSEP values and the number of principal factors of the PLS model are shown in Figure 2B. The optimal rank was determined as 14 and then the optimal PLS model (M10) was established.

**Figure 3:**
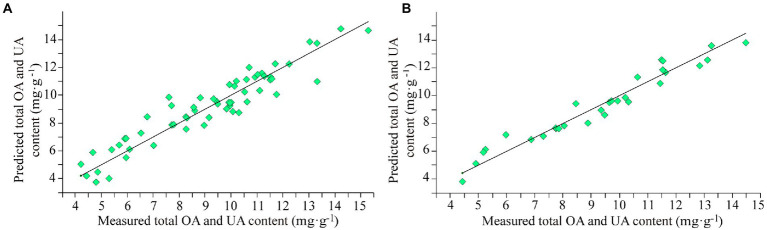
The results of model M10 for the total content of OA and UA. **(A)** Internal cross-validation and **(B)** external validation.

The internal cross-validation and external validation results of model M10 are shown in Figure 3. The modeling effect of model M10 was improved than model M7 with RMSECV of 0.87 mg·g^−1^ and *R*^2^ of 88.65% in internal cross-validation and RMSEP of 0.61 mg·g^−1^, *R*^2^ of 94.55%, RPD of 4.28 and bias of 0.0164 mg·g^−1^ in external validation.

### PLS-BP-ANN model

In the PLS modeling process, NIRS data was optimized and a strong linear correlation between the 14 principal factors extracted from the optimal PLS model and the content data of Chaenomelis Fructus was demonstrated. Therefore, the score data of the 14 principal factors was used as the input variables of the ANN model, the total OA and UA content determined by HPLC as the output variables, and a 3-layer BP-ANN established by the nntool toolbox of MATLAB software. The transfer functions from the input layer to the hidden layer and from the hidden layer to the output layer were set to be logarithmic S-type (logsig) function and linear (purelin) function. The learning function was the BP learning rules (learngd) and training function quantified conjugate gradient method (trainscg). The number of network training steps was set to 1,000; the goal error of the network was set to 0.001; the training speed of the network was set to 0.1.

**Figure 4:**
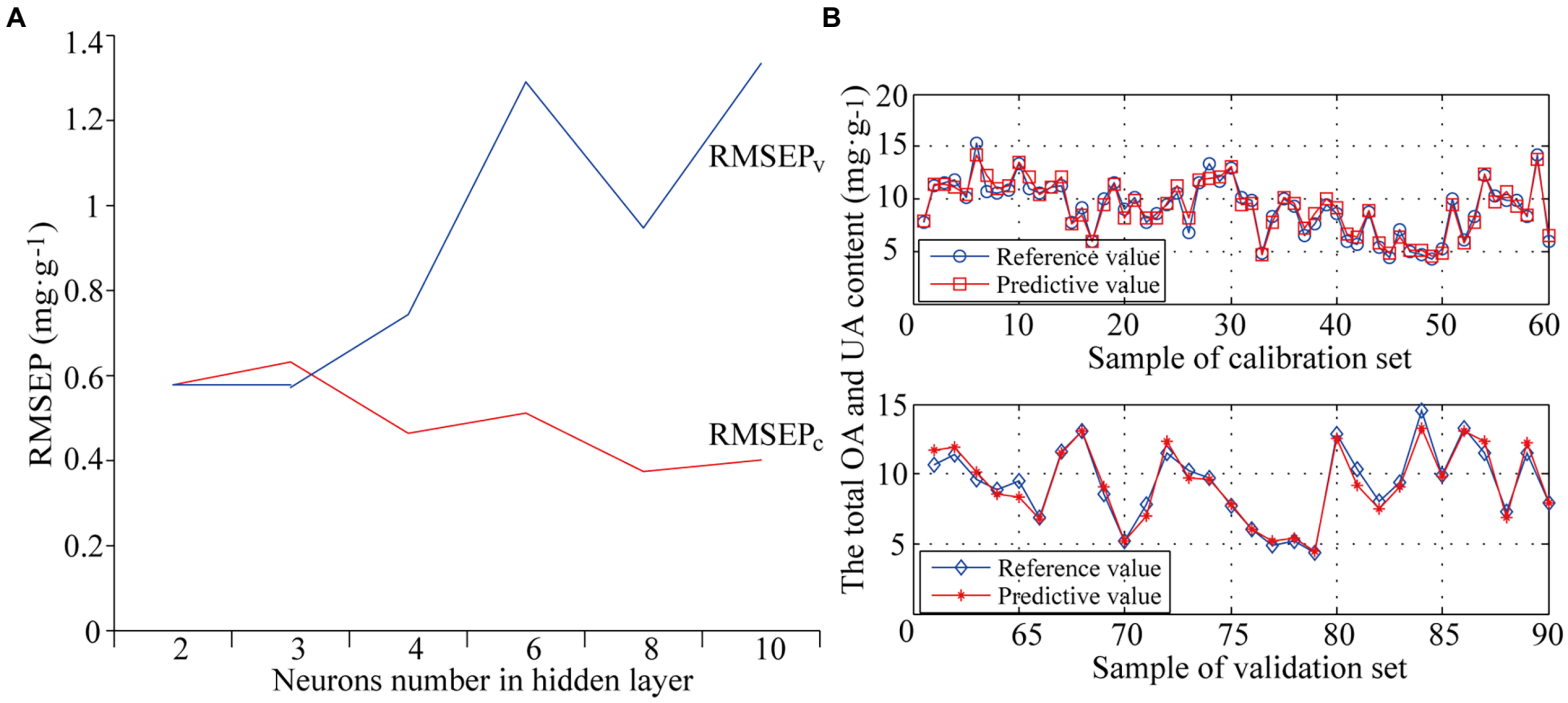
The optimization of neurons number in hidden layer **(A)** and prediction results of internal across validation and external validation of model M11 **(B)**.

The hidden layer is the layer between the input layer and output layer. In the structure of BP-ANN, the number of nodes in the hidden layer affects the calculation results of the model to a great extent. It is necessary to choose the best number of hidden nodes. Six ANN models were built with different numbers of neurons set in the hidden layer, 2, 3, 4, 6, 8, and 10. Models were evaluated by internal cross validation and external validation. The optimization results are shown in Figure 4. Figure 4A shows that RMSEP_v_ was significantly greater than RMSEP_c_ and the model was over-fitted with poor predictive ability when the number of neurons was above 3; RMSEP_c_ and RMSEP_v_ were very close and smaller with better modeling performance when the number of neurons was 2 or 3. For model simplification, the optimal number of neurons in the hidden layer was determined as 2 with the optimal PLS-BP-ANN model obtained by training. Figure 4B shows the prediction results of internal across validation and external validation of the optimal model (M11). The model was excellent with RMSEP_c_ of 0.58 mg·g^−1^ and *R_c_*^2^ of 95.06% in internal cross validation and RMSEP_v_ of 0.58 mg·g^−1^, *R_v_*^2^ of 95.29%, RPD of 4.66, and bias of −0.0624 mg·g^−1^ in external validation.

### Comparison and evaluation of models

The optimal PLS model (M10) in this paper was built through screening spectral pretreatment methods, characteristic spectrum, and the number of principal factors. On this basis, the score data of the first 14 principal factors was extracted as the input variables of BP-ANN with the number of neurons in the hidden layer set to 2 and the optimal PLS-BP-ANN model (M11) obtained by training. Compared to M10, M11 had smaller RMSEP_v_, greater *R_v_*^2^ and RPD, and significantly better validation effects with acceptable bias.

**Figure 5:**
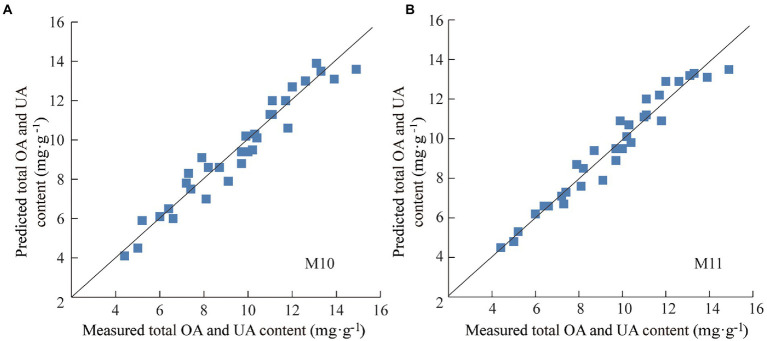
Prediction results for the total content of OA and UA of test set samples by model M10 **(A)** and M11 **(B)**.

To further determine the predictive ability and applicability of M10 and M11, practical application test was conducted using 32 batches of the test set samples that were not involved in modeling process. Specifically, the total OA and UA content of test set samples determined under the same HPLC conditions was used as reference value, and the NIR spectra of the test set samples were collected under the same conditions as modeling samples. The total content of OA and UA in the test set ranged from 4.4 to 14.9 mg·g^−1^, with an average of 9.5 mg·g^−1^ and a standard deviation of 2.7 mg·g^−1^, indicating that the test set was typical and within the application scope of modeled samples. The total OA and UA content of each test set sample was predicted by M10 and M11, respectively. The prediction results of the test set samples are shown in Figure 5 and Table 4. Both model M10 and M11 had strong predictive ability, in which M11 was significantly better than M10. Both the average relative deviation (ARD) and bias of M11 were smaller than those of M10, which indicates that model M11 had higher prediction accuracy and reliability. Model M11 was finally considered the optimal quantitative model for the total content of OA and UA in Chaenomelis Fructus.

**Table 4:** Statistic results of the test set.

Model number	RMSEP (mg·g^−1^)	*R*^2^ (%)	RPD	Bias (mg·g^−1^)	ARD (%)
M10	0.68	93.72	3.95	0.0498	6.3
M11	0.59	95.10	4.53	0.0387	4.8

The comparison between M10 and M11 indicated that the performance and predictive ability of the BP-ANN model were significantly better than that of the PLS model with the same spectral pretreatment, spectral waveband, and dimension reduction conditions. The practical application of the two models to the test set samples also proved that the BP-ANN algorithm basing on non-linear fitting has better modeling ability and higher prediction accuracy and precision than the linear regression algorithm.

## Conclusion

NIRS mainly reflects the absorption of overtone and combination peaks containing hydrogen bonds of C-H, O-H, and N-H. To reveal the relevant information embedded in the raw spectral data, the application of chemometric methods to build calibration models is the key to achieving qualitative or quantitative analysis. In this paper, the PLS method was first adopted to establish the NIRS quantitative model of the total content of OA and UA in Chaenomelis Fructus. The scoring value of 14 ranks obtained by PLS dimension reduction was taken as input variables of BP-ANN model, and then PLS-BP-ANN model was established. The model showed excellent performance and strong prediction capacity. This study indicated that NIRS combined with chemometric algorithms could be successfully used for rapid determination of the total content of OA and UA in Chaenomelis Fructus, which is of great help to the rapid quality evaluation and control of Chaenomelis Fructus. Compared to HPLC, the NIRS method has the advantages of simple pretreatment, fast analytical speed, high multisample processing capacity, no chemical wastes, which is more environmentally friendly. In the future, further studies should be carried out with the help of nondestructive acquisition methods like optic fiber probes to realize real-time and online measurements in industrial production. This research can be used as a reference for the rapid quality control of Chaenomelis Fructus and other Chinese medicinal materials during planting, processing and production.

## Data availability statement

The original contributions presented in the study are included in the article/Supplementary material, further inquiries can be directed to the corresponding authors.

## Author contributions

JM, LC, and BH developed the research hypothesis and the study design. BH provided financial support. MiL collected the samples and performed the chemical analysis. MinL performed statistical analysis. JM and LC performed NIR scanning, model development and wrote the manuscript. All authors contributed to the article and approved the submitted version.

## Funding

This work was supported by Research Project of Traditional Chinese Medicine of Hubei Provincial Health Commission (ZY2019Q013) and Science and Technology Program of Xiangyang, China (2019YL09).

## Conflict of interest

The authors declare that the research was conducted in the absence of any commercial or financial relationships that could be construed as a potential conflict of interest.

## Publisher’s note

All claims expressed in this article are solely those of the authors and do not necessarily represent those of their affiliated organizations, or those of the publisher, the editors and the reviewers. Any product that may be evaluated in this article, or claim that may be made by its manufacturer, is not guaranteed or endorsed by the publisher.
