# Supplementary material for: Rapid determination of the total content of oleanolic acid and ursolic acid in Chaenomelis Fructus using near-infrared spectroscopy
Source: Front Plant Sci. 2022 Sep 2;13:978937. doi: 10.3389/fpls.2022.978937 (PMC9478200; doi:10.3389/fpls.2022.978937)
Supplement: Supplementary file 1 [file Table_1.DOCX]

**Supplementary Table 1.** The results of OA and UA contents in modeling samples of Chaenomelis Fructus.

| Number | OA (mg·g^-1^) | UA (mg·g^-1^) | Total content of OA and UA (mg·g^-1^) |
| --- | --- | --- | --- |
| 1 | 6.8 | 0.9 | 7.7 |
| 2 | 9.8 | 1.4 | 11.2 |
| 3 | 9.1 | 1.6 | 10.6 |
| 4 | 10 | 1.5 | 11.5 |
| 5 | 10.3 | 1.4 | 11.8 |
| 6 | 8.8 | 1.4 | 10.1 |
| 7 | 13.4 | 1.9 | 15.3 |
| 8 | 9.3 | 1.4 | 10.7 |
| 9 | 9 | 1.5 | 10.5 |
| 10 | 9.5 | 1.5 | 10.9 |
| 11 | 11.7 | 1.6 | 13.3 |
| 12 | 9.3 | 1.7 | 11 |
| 13 | 9.1 | 1.5 | 10.6 |
| 14 | 8 | 3.1 | 11.1 |
| 15 | 9.7 | 1.7 | 11.4 |
| 16 | 8.3 | 1.4 | 9.6 |
| 17 | 7.6 | 1.3 | 8.9 |
| 18 | 9.5 | 1.8 | 11.3 |
| 19 | 5.1 | 2.6 | 7.7 |
| 20 | 8.3 | 0.8 | 9.1 |
| 21 | 4.6 | 1.4 | 6 |
| 22 | 8.5 | 1 | 9.5 |
| 23 | 7.7 | 2.3 | 10 |
| 24 | 10.3 | 1.2 | 11.5 |
| 25 | 7.9 | 1.1 | 9 |
| 26 | 5.3 | 1.6 | 6.9 |
| 27 | 9.2 | 1 | 10.2 |
| 28 | 7 | 0.8 | 7.8 |
| 29 | 6 | 2.6 | 8.6 |
| 30 | 8.3 | 1.2 | 9.4 |
| 31 | 5.5 | 5 | 10.6 |
| 32 | 2.6 | 4.2 | 6.8 |
| 33 | 10.7 | 0.9 | 11.6 |
| 34 | 12 | 1.3 | 13.3 |
| 35 | 10.6 | 1 | 11.6 |
| 36 | 10.5 | 1.2 | 11.7 |
| 37 | 9.5 | 3.5 | 13 |
| 38 | 10 | 3.1 | 13.1 |
| 39 | 8.9 | 1.2 | 10.1 |
| 40 | 7.7 | 0.8 | 8.5 |
| 41 | 9.1 | 0.9 | 9.9 |
| 42 | 2.1 | 3.2 | 5.2 |
| 43 | 1.9 | 2.9 | 4.8 |
| 44 | 6.2 | 2.1 | 8.3 |
| 45 | 7.7 | 2.3 | 10 |
| 46 | 4.7 | 3.1 | 7.8 |
| 47 | 8.4 | 0.9 | 9.3 |
| 48 | 10.2 | 1.3 | 11.5 |
| 49 | 7.7 | 2.5 | 10.2 |
| 50 | 5.1 | 1.5 | 6.5 |
| 51 | 5.7 | 1.9 | 7.6 |
| 52 | 8.7 | 0.8 | 9.5 |
| 53 | 9.1 | 0.7 | 9.7 |
| 54 | 5.8 | 2.7 | 8.6 |
| 55 | 5.5 | 0.4 | 5.9 |
| 56 | 7.2 | 0.5 | 7.7 |
| 57 | 5.1 | 0.6 | 5.7 |
| 58 | 7.8 | 1 | 8.8 |
| 59 | 5.5 | 0.4 | 6 |
| 60 | 4.4 | 0.6 | 4.9 |
| 61 | 4.8 | 0.6 | 5.4 |
| 62 | 4.2 | 0.2 | 4.4 |
| 63 | 6.4 | 0.7 | 7 |
| 64 | 4.3 | 0.5 | 4.9 |
| 65 | 4.4 | 0.3 | 4.7 |
| 66 | 4.9 | 0.3 | 5.2 |
| 67 | 4 | 0.4 | 4.4 |
| 68 | 2.6 | 1.6 | 4.2 |
| 69 | 4.7 | 0.6 | 5.3 |
| 70 | 11.4 | 1.5 | 12.8 |
| 71 | 9 | 0.9 | 10 |
| 72 | 9.1 | 1.2 | 10.3 |
| 73 | 5.6 | 0.5 | 6.1 |
| 74 | 5.7 | 2.4 | 8 |
| 75 | 7.2 | 2.2 | 9.4 |
| 76 | 4.7 | 3.6 | 8.3 |
| 77 | 13.2 | 1.3 | 14.5 |
| 78 | 11.1 | 1.2 | 12.2 |
| 79 | 9.1 | 1.2 | 10.3 |
| 80 | 6.8 | 3.1 | 9.9 |
| 81 | 12.1 | 1.2 | 13.3 |
| 82 | 9 | 1 | 9.9 |
| 83 | 8.8 | 1 | 9.8 |
| 84 | 8.8 | 2.7 | 11.5 |
| 85 | 7.4 | 0.9 | 8.3 |
| 86 | 6.6 | 0.7 | 7.3 |
| 87 | 10.5 | 1.1 | 11.5 |
| 88 | 12.6 | 1.6 | 14.2 |
| 89 | 6.9 | 1 | 7.9 |
| 90 | 5.5 | 0.5 | 6 |
| Mean | 7.7 | 1.5 | 9.2 |
| SD | 2.6 | 0.9 | 2.6 |
